# Latent space arithmetic on data embeddings from healthy multi-tissue human RNA-seq decodes disease modules

**DOI:** 10.1016/j.patter.2024.101093

**Published:** 2024-10-31

**Authors:** Hendrik A. de Weerd, Dimitri Guala, Mika Gustafsson, Jane Synnergren, Jesper Tegnér, Zelmina Lubovac-Pilav, Rasmus Magnusson

**Affiliations:** 1 School of Bioscience, Systems Biology Research Center, University of Skövde, 541 45 Skövde, Sweden; 2 Department of Physics, Chemistry and Biology, Linköping University, 581 83 Linköping, Sweden; 3 Department of Biomedical Engineering, Linköping University, 581 83 Linköping, Sweden; 4 Department of Biochemistry and Biophysics, Stockholm University, 171 21 Solna, Sweden; 5 Merck AB, 169 70 Solna, Sweden; 6 Department of Molecular and Clinical Medicine, Institute of Medicine, The Sahlgrenska Academy at University of Gothenburg, 413 45 Gothenburg, Sweden; 7 Biological and Environmental Science and Engineering Division, King Abdullah University of Science and Technology (KAUST), Thuwal 23955–6900, Saudi Arabia; 8 Unit of Computational Medicine, Department of Medicine, Center for Molecular Medicine, Karolinska Institutet, Karolinska University Hospital, L8:05, 171 76, Stockholm, Sweden; 9 Computer, Electrical and Mathematical Sciences and Engineering Division, King Abdullah University of Science and Technology (KAUST), Thuwal 23955-6900, Saudi Arabia; 10 Science for Life Laboratory, Tomtebodavägen 23A, 171 65, Solna, Sweden

**Keywords:** module inference, gene expression analysis, variational autoencoder, disease mechanisms, drug repurposing, latent space analysis, disease signal extraction

## Abstract

Computational analyses of transcriptomic data have dramatically improved our understanding of complex diseases. However, such approaches are limited by small sample sets of disease-affected material. We asked if a variational autoencoder trained on large groups of healthy human RNA sequencing (RNA-seq) data can capture the fundamental gene regulation system and generalize to unseen disease changes. Importantly, we found this model to successfully compress unseen transcriptomic changes from 25 independent disease datasets. We decoded disease-specific signals from the latent space and found them to contain more disease-specific genes than the corresponding differential expression analysis in 20 of 25 cases. Finally, we matched these disease signals with known drug targets and extracted sets of known and potential pharmaceutical candidates. In summary, our study demonstrates how data-driven representation learning enables the arithmetic deconstruction of the latent space, facilitating the dissection of disease mechanisms and drug targets.

## Highlights

• A VAE model can learn fundamental gene expression patterns from public data
• Such a model can be used to understand more limited samples of human disease
• We show this model to be useful to study independent diseases and suggest drugs

## The bigger picture

The human transcriptome is a highly complex system and is often the focus of research, especially when it fails to function properly, causing disease. Indeed, the amount of publicly available transcriptomic data has grown considerably with the advent of high-throughput techniques. Such special cases are often hard to fully dissect, since studies will be confined to limited data samples and multiple biases. An ideal approach would utilize all available data to learn the fundamentals of the human gene expression system and use these insights in the examination of the more limited sample sets relating to specific diseases. This study shows how a neural network model can be created and used to extract relevant disease genes when applied to limited disease datasets and to suggest relevant pharmaceutical compounds. Thus, it presents a step toward a future where artificial intelligence can advance the analysis of human high-throughput data.

## Graphical abstract

**Figure:**
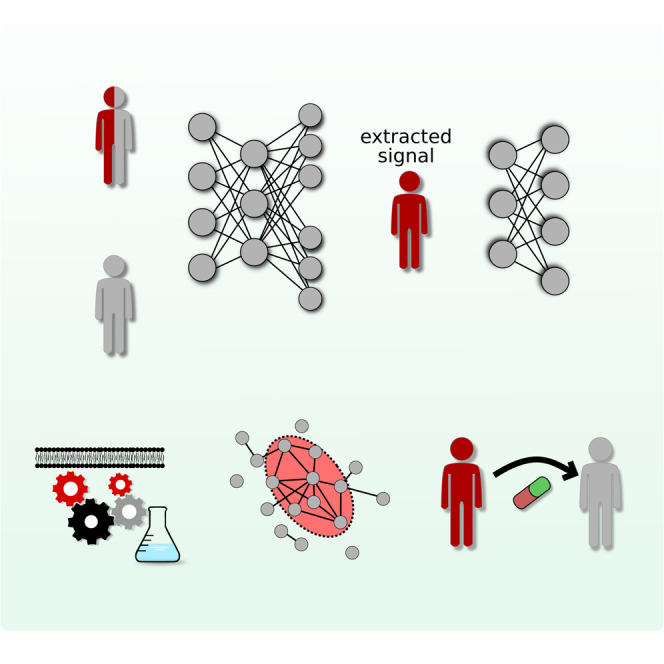

## Teaser

The computational analysis of gene expression data is often greatly limited by sample size. Here, the authors train an AI model to learn the fundamentals of gene expression from large sets of healthy human transcriptomics data. Leveraging the learned features of this model, the authors extract sets of important genes from independent data and use them to suggest relevant pharmaceutical compounds. This work presents a novel approach to utilize existing data in the analysis of more limited samples.

## Introduction

The human transcriptome reflects a network of genomic processes that control cellular functions. These processes manifest as networks of interconnected groups of functionally related genes, and this modular topology has long been utilized to study fundamental cellular biology.^1^ Various network disruptions, such as genetic changes and dysregulation, disrupt their proper function. While these disease factors may span the entire human interactome, they do not occur randomly but instead tend to co-localize in sets of functionally related genes referred to as modules.^2^

Studying such disease modules is essential for understanding pathophysiology, as holistic and mechanistic insights can be used to predict potential drug targets or biomarkers for disease prognosis.^3^ Correspondingly, multiple computational methods are now available for understanding how disease changes co-occur in the gene regulatory network,^4^ with a substantial portion of work revolving around data mining. These data may, for example, measure gene expression.^2^^,^^5^^,^^6^ In the case of complex diseases, the exact pathophysiologies at an intracellular level have arguably remained inconclusive.^6^ How to extract and interpret modules remains an open question.

The broader field of computational analysis of complex diseases has long struggled to find universally applicable methods that can distill knowledge from a broad selection of datasets. Complex diseases are inherently multi-factorial, with many subtypes and differing characteristics. The amount of available transcriptomics data from healthy sources dwarfs that of most specific diseases,^7^^,^^8^ and the significant advancement in our ability to model complex systems using large datasets with neural network models^9^ has paved the way for a new era of computational analysis. Yet, studies constrained by limited sets of disease-related tissue material struggle to capture the essence of a disease change. It follows that insights often fail to generalize, such that conclusions are specific to the limited material at hand.^10^ While transcriptomes reflect deep biology and disease-related perturbations, the limited number of disease samples and the challenge of identifying and using modules constitute a remaining hurdle slowing the discovery of novel biomarkers and drug candidates.^11^

To what extent have recent advancements using neural networks been useful in mitigating the challenges associated with modules and a limited number of disease-relevant datasets? Indeed, deep autoencoders—neural networks designed to compress data to a significantly smaller set of variables—have been found to encode sets of functionally related genes using the same variables, known as hidden nodes, when applied to gene expression data.^12^^,^^13^^,^^14^ However, these findings primarily suggest that autoencoders learn modularity to compress expression data rather than providing an approach to extract the most relevant latent structures for a specific disease state. In other words, how to extract and utilize the encoded structures remains an open question. Moreover, dissecting what input variables are encoded in which latent variable is not straightforward, despite recent progress in explanatory AI.^15^ Furthermore, suppose the autoencoder is not variational in the latent space. In that case, the encodings in the compressed space do not necessarily have a continuously meaningful biological representation, leaving perturbations as a means of model analysis flawed.^16^

Drawing inspiration from foundational models in machine learning,^17^ we ask whether the learned representation from training a machine learning model using rich and extensive data from domain A can be used to analyze data from domain B. In our setting, we explore whether a deep variational autoencoder (VAE) trained on healthy human RNA sequencing (RNA-seq) gene expression profiles (rich data domain A) from the Genotype-Tissue Expression project (GTEx) resource^7^ can capture the fundamentals of human gene regulation. If so, can such a system-wide model be generalized to explain disease data, i.e., sparse data domain B? Interestingly, we found that after training, the latent space of the VAE represented biological structures.

Moreover, we could extract hierarchical biological representations in the latent space, ranging from resolving cell types to capturing transcription factor (TF)-target interactions. Simultaneously, we observed that known disease features from genome-wide association studies (GWASs) are aggregated along the principal components of the latent space. Importantly, we also found this model to predict unseen gene expression data from The Cancer Genome Atlas (TCGA).^18^ These findings motivated us to design a generally applicable method for extracting modules that third parties, even those with small disease sample sizes of data, can easily use. We then showcased this method on a compendium of 25 human disease datasets and found highly disease-relevant modules. Most gene sets exhibited a higher enrichment of associated disease genes than the top differentially expressed genes (DEGs). This suggests that our approach identifies novel and intriguing disease-related genes beyond those with a clear disease signal in the data and yields more relevant disease-related sets of genes compared to the gold standard differential gene expression analysis of transcriptomic data. Finally, investigated disease gene sets showed significant enrichment of drug targets strongly associated with relevant pharmaceutical components for the analyzed diseases.

## Results

**Figure 1:**
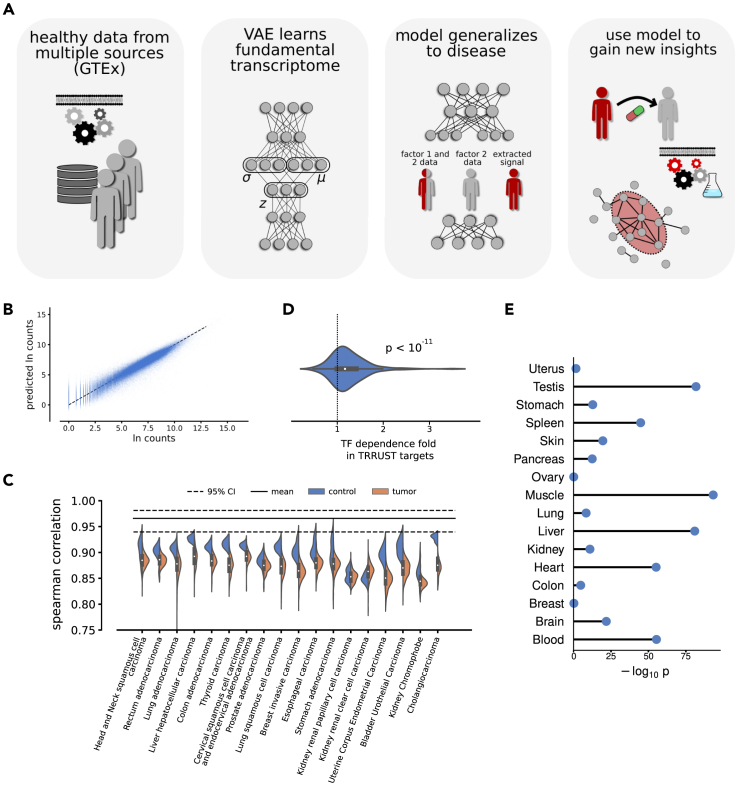
Model construction and testing (A) The workflow. The variational autoencoder (VAE) model was implemented as a feedforward VAE. We trained this model exclusively on healthy RNA-seq data and found it to generalize to disease data. Using simple arithmetic operations in the latent space, we extracted specific disease vectors and used these vectors to identify disease modules and suggest suitable drug candidates. (B) The model performance on test data is shown as blue dots. There was a clear similarity between the input and the output of the VAE. (C) The VAE was applied to independent data from TCGA, and the Spearman correlations over each transcriptomic profile were calculated. The respective correlation of the GTEx data from (B) is plotted as a black line. In comparison, the independent data performed slightly worse, with healthy data (blue) performing slightly better than data from human tumors (orange). All correlations were highly significant. (D) We tested the responsivity of known TF-target regulations by increasing a TF on the input level and measuring the changes on the output level of the VAE. The mean changes in known targets normalized by the mean of all other genes on the output level were plotted, showing the distributions for the TFs. Most values were >1, indicating a stronger association in the model (binomial test *p* value shown). (E) We tested if cell types were encoded in the latent space and compared top-associated genes of 16 tissues in the data to tissue-gene associations in the TISSUES 2.0 database using Fisher’s exact test. Shown are the nominal −log_10_ probabilities of the observed overlap or greater given H0: the sets are independent.

First, a VAE is trained to compress human gene expression data to test the hypothesis that the learned representations in the latent space may embed biological processes. We continue by analyzing learned structures of the latent space and finding biological patterns ranging from cell type to gene-gene interaction-specific structures. We show how elementary arithmetic operations on this latent parametrization of gene expression can be used to extract disease-relevant modules when studying independent expression data from 25 disease datasets. The respective modules outperform differential gene expression analysis in predicting known disease genes in this analysis. Finally, we demonstrate how the parametrization in the VAE latent space, when applied to disease-related expression profiles, can be used to recommend pharmaceutical compounds, suggesting our approach is important in drug repurposing studies (Figure 1A).

### Training a VAE using healthy RNA-seq profiles generalizes to unseen transcriptional disease profiles

The human transcriptome is a complex network of expressed genes that regulate a wide array of cellular functions and processes. Our goal was to identify a meaningful representation of this system that could serve as a foundation for further biological studies. To achieve this, we developed a data-driven approach using a deep VAE to compress transcriptome data, operating under the rationale that this compression would encode underlying biological structures within the latent space. We implemented the VAE (Figure 1A) as a fully connected feedforward neural network, with three hidden layers of 128, 2 × 64, and 128 nodes, respectively, where the middle variational layer contained 64 mean and standard deviation node pairs. Next, we used bulk RNA-seq data from the 30 distinct healthy tissues from the 948 donors in the GTEx, randomly divided into 11,258 training, 4,362 validation, and 1,762 test samples, which were unused in model training. In total, we used 16,819 genes from the GTEx data (see experimental procedures). We evaluated the data loss of the trained model after compression using the test data estimated with Spearman’s rank correlation for each test expression profile. We found a mean correlation of 0.96 (95% confidence interval 0.94–0.98) (Figures 1B and 1C). Furthermore, we observed that the contribution to the total loss from the Kullback-Leibler (KL) divergence was approximately 0.4016 per latent variable, while the reconstruction loss, in this case the mean squared error, per input variable was 0.0003, resulting in an evidence lower bound (ELBO) per data point at −0.4018.

Knowing that the model could faithfully re-create healthy human material, we next aimed to validate the learned compression on independent disease data. To this end, we used case-control datasets from The Cancer Genome Atlas as processed by Wang et al.,^19^ noting that cancer is one of the most heavily perturbed disease systems. We ran each tissue and respective tumor gene expression profile through the trained VAE and calculated Spearman’s rank correlation between model input and output for each transcriptomic profile. Strikingly, we found strong correlations before and after compression for all datasets (mean Spearman’s ρ = 0.87, Figure 1C), with the healthy controls (mean ρ = 0.90) performing slightly better than the tumor data (mean ρ = 0.87). In other words, the VAE, which had been trained to compress exclusively expression data of healthy material, could be used to represent and compress unseen transcriptional disease profiles. In comparison, in a previous study, we built a feedforward neural network to predict transcriptomic profiles from TF expression, for which we reported an out-of-sample prediction on tumor data with 80% predicted variance.^9^

### Rich biological multi-scale representation in the learned latent space

During the training of an autoencoder, data structures of varying resolutions are known to be encoded into the latent space.^12^ Such encodings can be viewed from an image analysis analogy, where structures ranging from basic shapes to complex features such as faces manifest in the hidden layers of a VAE. We aimed to search for such biological structures where the resolution would range between gene-gene interaction and high-dimensional structures such as pathways. The data structures, although nontrivial to extract, are exciting since they potentially hold biological insights unbiased by *a priori* biological assumptions. We, therefore, aimed to examine these biological structures further.

The training data from the GTEx contained bulk RNA-seq from 30 distinct healthy human tissues divided into 54 subcategories, and we hypothesized that the differences between tissue types are the most significant source of variation in the training data. We therefore examined whether tissue-specific gene sets could be extracted from the latent space. To this end, we downloaded the knowledge channel of the TISSUES 2.0 database containing manually curated known associations between gene expression and human tissue types.^20^ We matched the 30 distinct tissue types in our data to the tissue types present in TISSUES 2.0, considering only literal matches, i.e., “Muscle” to “Muscle” and so forth. Next, we filtered the matches further by requiring at least 100 tissue-gene associations in the database and at least 10 samples for each tissue type in our test set, resulting in 16 distinct tissue types.

To extract tissue-specific gene sets, we employed the strategy described in the experimental procedures (specifically Equations 1, 2, 3, and 4). We analyzed the top 500 ranked genes for each of the 16 tissue types for tissue-specific enrichment using a nominal Fisher’s exact test. Notably, we found major enrichments for 14 of the 16 tested tissue types, with exceptionally high enrichments for the heart, liver, muscle, and testis (Figure 1E). A possible explanation for the poor performance in some tissues relates to limited sample sizes in the training data and the potential for the model to improve as access to more healthy tissue data increases. Furthermore, since tissues consist of several cell types, we present a more extensive analysis in Table S1, which includes all statistically significant cell type matches.

We next analyzed biological pathways to assess if more fine-grained-resolution cell structures were encoded in the latent space. Since there is arguably no approach to directly extract learned structures from the latent space and to analyze the latent structures, we decided to perform a linear principal-component analysis (PCA) on the compressed test data from the GTEx dataset. The rationale of using a PCA was that the latent space is not designed to be orthogonal and that features represented in this space are not necessarily encoded along one single node. We then augmented the latent space compression of the data by increasing the activations along each principal component (see experimental procedures). Next, we considered the top 500 changing genes for each principal-component perturbation and calculated the overlaps with genes associated with KEGG pathways. We found 3,594 statistically significant principal-component-KEGG pathway overlaps (median odds ratio [OR] = 30.8, 50% on the interval [17.3, 60.5]) using a Bonferroni-corrected Fisher’s exact test, using the 16,819 genes of the model input as background. This suggests that biological processes are represented in the latent space.

We hypothesized that the training data variance had several sources, ranging from system-wide patterns, such as cell type, to more fine-grained resolutions, such as the state of intracellular pathways. Prompted by our results regarding the KEGG pathways, we continued to study individual gene-gene interactions. We have previously shown how neural networks can use TFs to predict target gene expression,^9^ and we herein used the same approach to associate TFs with target genes. In other words, the fact that both TFs and target genes were clearly defined in the input and output layers of the model enabled a more direct approach than studying the principal components of the latent space. For each TF, we increased the expression in the test data with 5 standard deviations and measured the gene response on the output level, defined as the absolute gene change from the TF increase. We measured the mean of this response for known target genes in the TRRUST^21^ database and found on average 24% higher levels than for the genes not registered as known target genes in TRRUST, with 140 of 190 TFs having target genes with higher variance (binomial test *p* = 2.4 × 10^−11^, Figure 1D). Even though the increase was lower than the 70% found in our earlier work,^9^ we note how that model was specifically designed to capture TF-target regulations.

### Known disease genes aggregated along different principal components in the latent space

With results indicating that biological mechanisms from the healthy data were incorporated into the learned latent space structure, we sought to test this phenomenon in relation to known disease associations. We, therefore, downloaded the GWAS catalog^22^ and again augmented the principal components of the compressed latent space using the test data. For each principal component, we calculated the overlap of the top 500 augmentation-responding genes with genetic variations associated with disease in the GWAS catalog.

Using a Bonferroni-corrected Fisher’s exact test with a background of the 16,819 genes used as model input, we found 311 significant disease-principal component pairs. Moreover, we found 2,917 nominally significant pairs, a 3.4-fold increase in what would be expected under the null hypothesis of random overlap (expected 857.6 of 17,152 disease-principal component pairs at a 0.05 rejection rate).

### Algebraic manipulation of identified case-control vectors in the latent space extracts highly disease-relevant modules for 25 disease studies

A core advantage of the VAE is the inherent continuous encoding of representations in the latent space. This representation of data structures has allowed earlier work in computer vision to show how images can be manipulated along an axis that outputs a continuous change in an encoded feature.^23^ Here, we explore whether an analogous operation could be translated into biological insights using gene expression data. Given a vector relevant to a specific disease, this vector could be used to extract relevant disease genes. Since GWAS genes were found to be enriched in certain vectors along the latent space structure, we decided to search for a direct approach to extract clusters enriched with known disease genes from the latent space. To this end, we downloaded all relevant case-control studies of complex diseases from the Expression Atlas database (see experimental procedures for inclusion criteria). Moreover, we also included five case-control studies used in our latest study on module inference,^24^ bringing the total number of disease sets to 25.

**Figure 2:**
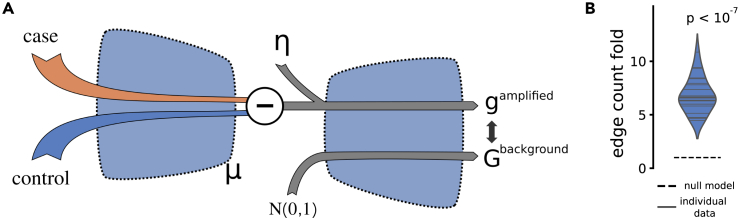
Highly modular gene sets could be extracted (A) We devised an approach to extract relevant groups of genes from factorial data, i.e., data containing disease case and control samples. These two factors were compressed into the latent space, and the respective mean difference was calculated, denoted as the disease vector. Next, the disease vector signal was amplified with a factor η and decompressed. The decompressed values were then compared to decompressed random, normally distributed data. (B) For the 25 studied disease datasets, we used our algorithm to extract sets of genes and used the STRING PPI network to calculate the enrichment of known interactions between these genes compared to the expected number of interactions. All gene sets had highly significant increases in STRING interactions between them, compared to the STRING background, with the increase typically being 5-fold or more. The *p* value shows the probability of all 25 samples being greater than the null model using a binomial test.

We hypothesized that the difference between two conditions could manifest as a vector in the latent space activations, which prompted us to arithmetically extract the differences in the latent space as opposed to the PCA-based search. In other words, for each disease, we searched for a vector in the latent space that corresponded to the respective transcriptomic changes. To derive such latent vectors, we compressed the case and control samples separately, and for each node in the latent space, we calculated the average difference between the two conditions. We referred to this vector as the disease vector and amplified the latent space signal 3-fold along the disease vector. Notably, VAEs by definition have a probabilistic representation layer that results in a continuous representation of data in the latent space. This feature enables amplification of node signals in the probabilistic layer to translate into information on disease-relative gene changes after decompression. To identify the impact of the amplification of the disease-relevant vector in the latent space, we decompressed the data that were augmented along the disease vector and, as a comparison, generated 1,000 random, normally distributed latent space variables and subsequently decompressed these profiles to the complete transcriptomic profiles. Next, we computed the rank for each gene in the decompressed disease vector in relation to the 1,000 random reference profiles. The method is further described in the experimental procedures, and a schematic representation can be found in Figure 2A. Modules are often defined as sets of genes that share a biological function and, therefore, are densely connected on a gene interaction graph. To analyze how the inferred output genes co-occurred from a network perspective, i.e., to what degree these sets formed modules, we applied our method to our compendium of 25 disease expression sets. We independently mapped the top 500 output genes from each of the 25 disease datasets to the STRING protein-protein interaction (PPI) network and calculated the edge enrichment for each gene set using the STRINGdb R package.^25^ All gene sets were highly modular regarding increased edges between the genes within each module (median fold of expected edges 6.1, Figure 2B). We next filtered our generated gene sets to contain only the largest component connected in the STRING PPI.

### Extracted modules were more enriched with disease-related genes than with the respective observed DEGs

**Figure 3:**
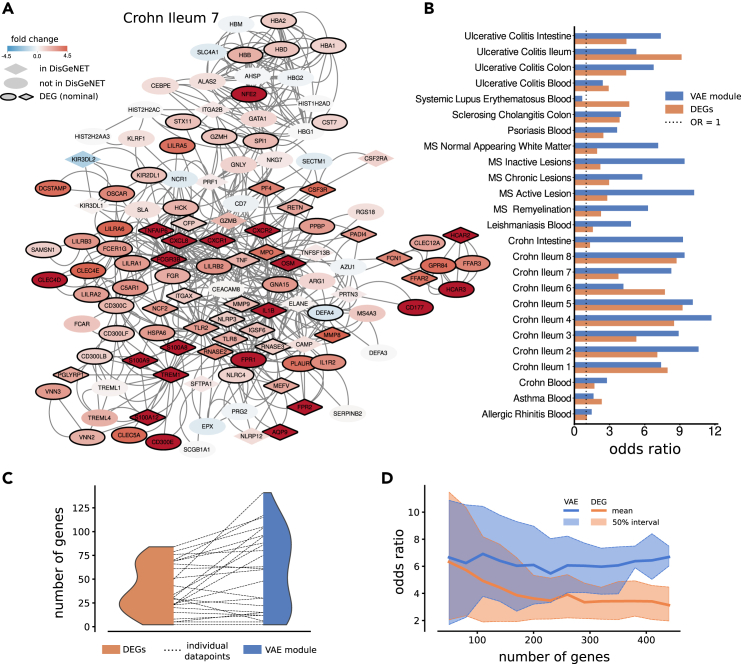
Modules were significantly enriched for known disease genes (A) An example of a module of size 500 genes extracted using RNA-seq data from samples of the ileum of Crohn’s disease patients and healthy controls. As can be seen, several genes were not differentially expressed in the data but were associated with Crohn’s in DisGeNET. (B) The OR of Fisher’s exact test for overlap with DisGeNET genes is shown for the VAE module and the respective top DEGs. The modules were extracted with a size of 500 genes, which is the default setting. Strikingly, the VAE-derived genes are more relevant for each disease than the corresponding top DEGs in the data. (C) The distribution of DisGeNET genes in the top DEGs and in the equally sized respective modules is shown for the diseases. The increase in the number of genes for each disease is shown by the dotted line between the two distributions. (D) The mean overlap of the VAE modules and the top DEGs with the DisGeNET genes for all diseases as a function of the number of genes showed a better performance for the VAE modules. Interestingly, the modules seemed to be stable in terms of OR with respect to module size, suggesting our approach to be robust.

Knowing that the output gene sets were highly modular, we henceforth considered the sets as modules. To test the relevance of these modules with respect to the respective diseases, we next compared them to the corresponding known disease-associated gene sets in the DisGeNET database.^26^ We found clear enrichment of disease-associated genes across next-to-all diseases, including genes that were not found to be differentially expressed in the data source (Figure 3A). For each module, we again performed a Fisher’s exact test for enrichment of known associated disease genes and found striking enrichments of overlapping genes, typically with an OR between 4 and 8 (Figure 3B).

We next compared the number of disease-associated genes from the DisGeNET in the modules with the occurrence of DisGeNET genes among top DEGs and found the number of genes to be larger in the modules in most diseases (Figure 3C). Specifically, we used the DEG analyses accompanying the expression data from the Expression Atlas, and for the separate multiple sclerosis (MS) datasets we used the DEG analysis provided by Elkjaer et al.^27^ First, we filtered the DEGs to contain only genes in the STRING database and removed the DisGeNET disease-associated genes not present in the DEG data to ensure genes were drawn from the same pool as the modules.

Moreover, we used Fisher’s exact test between the equally sized set of top DEGs and the corresponding DisGeNET disease-associated gene sets to test for enrichment. In 20 of 25 cases (Figure 3B), the modules derived from the latent space using our trained model had a higher OR of overlap enrichment than the corresponding number of DEGs (binomial test 20 of 25: *p* =0.002). We also analyzed the impact of varying model sizes from 50 to 450 included genes and found the performance of our method to be surprisingly robust to module size. Furthermore, we found the modules of varying sizes to consistently outperform the DEG analysis in terms of DisGeNET overlap (Figure 3D). In other words, the modules extracted using our VAE were a more relevant foundation for disease analysis of factorial data than the gold standard technique, the DEG analysis. The test statistics for the VAE overlaps are found in Table S2.

To assess the contribution of the VAE in relation to the VAE used in tandem with the extraction of the largest connected component in our approach, we continued by comparing our results to the largest connected component of the DEGs. We found that our approach outperformed the largest connected component of the DEGs in 15 of the 25 disease sets. Moreover, the ORs of the VAE approach on average gave an increase of 50% compared to taking just the largest component of the DEG set ($\mu_{VAE}=6.38$, $\mu_{\mathrm{DEG}\;largest\;component}=5.25$, one-tailed paired Student’s t test, *p* = 0.025, degrees of freedom = 24). This test suggests that the VAE extraction adds biological relevance beyond gene topology. Furthermore, the increase in disease relevance showcases the overall importance of gene network topology in the interpretation of disease gene expression changes.

We recently published a study on how combining several module inference approaches greatly improves performance when extracting relevant gene sets from disease data.^6^ We therefore sought to characterize the genes extracted by our approach, in relation to the DEGs. For each disease we calculated the number of genes from the extraction using our VAE that were also present in the largest connected component in the DEG set. On average, 14% of the genes identified by the VAE method were present in the DEG set (>50% of the diseases on the interval [0%, 26%]). In fact, eight diseases had no overlap between the VAE and the DEG sets, and we believe this, together with the fact that next-to-all VAE sets were enriched with disease genes, to suggest that the VAE approach extracts a more generalized disease pattern than what can be inferred from the particular disease set.

We also sought to test how our VAE approach performed in the identification of DisGeNET genes compared to alternative module inference approaches. In our previously published study of module inference methods,^6^ the method Clique SuM^28^ performed the best. We decided to compare our approach to Clique SuM and applied it to our compendium of 25 disease datasets using the implementation in the MODifieR software package.^29^ Studying the ORs of extracted genes that were in the DisGeNET, we found our VAE approach to display on average 22% higher ORs ($\mu_{VAE}=6.38$, $\mu_{\mathrm{DEG}\;largest\;component}=5.85$, one-tailed paired Student’s t test, *p* = 0.21, degrees of freedom = 24), while the VAE approach had higher ORs in 12 of 25 disease datasets (Table S3). The performance of different methods can vary based on factors such as the network topology of disease-causing elements, the choice of gold standard, and specific data properties. Considering these variables, we assert that our method is comparable to the top performer in our previous work, where we benchmarked various module inference methods for extracting disease-relevant genes.

### Underlying structures in the latent space restrict the modes of analysis

**Figure 4:**
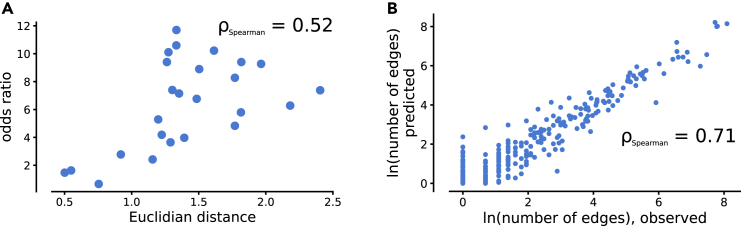
High performance is connected to defined areas of the latent space (A) We found the Euclidean distance between the case and the control vectors in the latent space to be well correlated with the observed enrichment of known disease genes on the output level. (B) We trained a regression model to predict the number of edges on a PPI network based on random vectors in the latent space and found a clear relationship.

We next asked how the vector amplitude in the latent space translates to meaningful biological interpretations on the decoded level. Starting with the analysis of disease module prediction based on case-control data (Figure 3), we hypothesized the differences between case and control samples to indicate disease signal and, in extension, method performance. We thus calculated the mean Euclidean distance between the compressed case and control vectors in the latent space. We found this metric to be well correlated with the previously presented ORs of disease gene enrichments (Figure 4A, Spearman’s ρ=0.52,p=0.008).

We also asked if all vectors in the latent space correspond to an interpretable set of genes, and to this end, we generated 100,000 random latent space profiles. Typically, the vectors in the latent space that capture transcriptomic changes between disease and healthy state have, on average, low activation values compared to vectors of a compressed whole transcriptome. We believe this change in amplitude to originate in the disease changes to be a subpart of the broader transcriptome signal. To mimic these low activation values, we sampled the activation values from a standard normal distribution with μ = 0 and σ to be a random number from the uniform distribution between 0.01 and 0.4. Next, we translated the random latent space profiles into a module, as described under “module extraction” in the experimental procedures, but without the augmentation of the latent space activations, and calculated the number of edges in the output module. We found most sets to have zero or few PPIs between them. Furthermore, we trained a regressor model to predict the number of PPIs. We found the performance on a separate test set of 10,000 profiles prepared similarly (Figure 4B), suggesting the modularity to be encoded within a well-defined area of the latent space.

### Enhancing the disease vectors suggests suitable pharmaceutical compounds

Having studied how the arithmetic extraction of disease-associated vectors in the latent space carried information on disease-associated genes, which arguably allows for further studies of pathophysiology and pathogenesis, we next tested how this parametrization could be used to predict suitable pharmaceutical compounds. Analogous to our study of GWAS genes, we analyzed the top 500 genes associated with each principal component of the latent space. We then iteratively compared each set of 500 genes to the known targets of all drugs with more than 10 known target genes in the DrugBank database.^30^ Specifically, for each of the 64 sets of 500 genes, we performed 264 Fisher’s exact tests to enrich drug targets, using the same background as in the previous tests. We found 59 of 64 gene sets to have more nominally significant associations than what would be expected by random chance (expected 32, $P_{binom}=4.6\times 10^{-13}$).

We continued the analysis by testing if we could use the model to suggest suitable drug candidates to the specific case-control transcriptomics sets in our disease compendium. We did this by matching the top genes in the decompressed output of the respective disease vectors to the known targets of the pharmaceutical compounds in DrugBank. In other words, we tested if we could extract potential pharmaceutical compounds to aid drug repurposing by associating the specific disease vector in the latent space to pharmaceutical compounds via the VAE decoder. While there is not necessarily a direct link between gene expression changes and causative disease changes, we found that the top-ranking drug suggestions for each disease were strikingly relevant. Here, we present five cases, while the ranked pharmaceutical compounds for all tested diseases are found in Table S4.

#### Case 1: MS

For the MS data presented in Elkjaer et al.,^27^ labeled “MS Active Lesion” in Figure 3, the top drug suggestions were muromonab, previously tested for MS,^31^ and ibrutinib, a Bruton’s tyrosine kinase inhibitor (BTKi), which are both highly relevant in MS research.^32^ The third-ranked drug was daclizumab, which has been approved as a treatment for MS^33^ but retracted due to adverse side effects. The fourth was zanubrutinib, another BTKi compound, and the fifth was alemtuzumab, which is currently used as treatment for MS.

#### Case 2: Crohn’s disease

We next tested the approach on Crohn’s disease, labeled “Crohn Ilium 1” in Figure 3.^34^ The top two matching compounds were zinc and zinc acetate, and zinc deficiency has been associated with poor clinical outcomes in people with inflammatory bowel diseases.^35^ The third compound was dilmapimod, a p38 mitogen-activated protein kinase (MAPK) inhibitor with known anti-inflammatory effects^36^; the fourth was glucosamine, which has been used in cases with osteoarthritis; while the fifth was VX-702, another p38 MAPK inhibitor.

#### Case 3: Systemic lupus erythematosus (SLE)

Analyzing the data in Hung et al.,^37^ labeled “Systemic Lupus Erythematosus Blood” in Figure 3, we found the top predicted drug to be gemcitabine. This chemotherapeutic agent has been reported to, albeit rarely, trigger SLE.^38^ We note that our approach does not consider the direction of gene dysregulation in relation to the suggested pharmaceutical compound. In fact, in the top five predicted compounds, three are primarily used as chemotherapeutic agents (gemcitabine, enzastaurin, and sunitinib), which we speculate to be related to the anti-nuclear immunological response of SLE. The remaining two drugs were fostamatinib, a tyrosine kinase inhibitor used in immune thrombocytopenia, and cladribine, a selective lymphocyte-depleting agent that is primarily used in the treatment of MS. We note, however, that cladribine has been tested as an agent against SLE.^39^ The case study of SLE is of interest as it was one of only a few cases that were not significantly enriched for DisGeNET genes. Yet, these results suggest a relevant disease signal in the module output.

#### Case 4: Psoriasis

In this case, we analyzed compounds predicted to be associated with the autoimmune disease psoriasis.^40^ Notably, copper, zinc, and zinc acetate were all among the top five predictions. Increased serum copper levels and decreased serum zinc levels have been linked to psoriasis, and there have been suggestions that normalizing these levels could be a potential treatment strategy for psoriasis patients.^41^ In addition, we identified the nonsteroidal anti-inflammatory drug (NSAID) diclofenac, which has been studied in relation to psoriasis.^42^ The fifth predicted compound was methylaminophenylalanyl-leucyl-hydroxamic acid, known to target neutrophil collagenase according to the BindingDB database.^43^

#### Case 5: Leishmaniasis

The fifth case involved an analysis of data from patients with leishmaniasis,^44^ a parasitic disease caused by protozoan infection that leads to the formation of skin ulcers. Among the top five predicted drugs, we again identified compounds linked to immune system modulation, including alemtuzumab, gemtuzumab, and the tumor necrosis factor inhibitor etanercept. Interestingly, the analysis also highlighted foreskin fibroblasts and foreskin keratinocytes as top candidates. This finding is intriguing, as it aligns with the pathology of leishmaniasis, where skin tissue involvement and immune response are critical in the disease’s progression and the body’s defense mechanisms.

## Discussion

Here, we have presented how a VAE can learn to represent the fundamentals of transcriptomics associated with human tissues. We show how this data-driven approach generalizes from exclusively healthy material to disease-affected domains, making representation learning practical. Moreover, we demonstrate how to extract biologically relevant insights from the latent space, thus adding broader value for biomedical research. We show how the latent vectorization of gene expression data can be utilized so that arithmetic functions can extract important disease signals. Using data from 25 independent disease datasets, we extracted sets of disease-relevant genes, herein referred to as disease modules, and compared them to previously known disease genes. All but one of the modules were significantly enriched for known disease genes, and notably, 20 of 25 cases outperformed the corresponding top DEGs. Furthermore, we show how these modules can be used to predict drug candidates with high therapeutic potential from approved and registered pharmaceuticals.

We hypothesize that the model’s performance originates from the encoded biological structures embedded in the latent space during training. Although the VAE was trained using exclusively healthy human gene expression profiles, the model could still explain previously unseen disease patterns. In addition, there is no fully established approach to extracting the encodings of the latent space, and our analyses of cell type, pathway, and TF binding mechanisms are limited. However, we claim that the model represents important transcriptome processes such that it can be used to understand at least a substantial set of human diseases.

Nevertheless, the model could potentially be improved. The GTEx data we used are limited to <20,000 samples; arguably, most variation stems from different cell types. We also note that others have found cell type to be the predominant parameter for latent space representation.^45^ While there are several other sets of massive gene expression datasets,^7^^,^^46^ we believe using the GTEx consortium data is a key strength of our analysis when applied to independent disease data. Furthermore, we note that the gold standard, i.e., the DisGeNET,^26^ used herein is not a complete set of relevant disease genes. Instead, all genes taking part in the generated modules are of interest from a mechanistic and a biomarker perspective.

Herein, we utilized the generative capabilities of the VAE to identify genes that are relevant to the differences observed between disease-affected individuals and controls in independent RNA-seq data. Specifically, we extracted genes associated with disease-related vectors that we identified directly from the VAE’s latent space. For this extraction to be meaningful, it is crucial that shifts along the disease vector correspond to valid biological functionality. The continuity and smoothness of a VAE’s latent space are particularly important in this context, as a standard autoencoder would likely be more prone to scenarios where nearby points in the latent space do not correspond to biologically related outputs. This consideration is also applicable to other generative AI models, such as generative adversarial networks (GANs), normalizing flows, and diffusion models.

This approach is part of a larger paradigm shift, where the availability of big biological data coupled with the potential of deep learning allows for complex biological features to be modeled without relying on what are often greatly limited datasets. Way et al.^13^ and Dwivedi et al.^12^ have recently shown autoencoders applied to gene expression data to learn cell functionalities, incrementally increasing with model depth. This work contrasts with that of Way et al. and Dwivedi et al., who both used independent machine learning models to extract specific encoded structures from latent layers, in that our method is generally applicable with the end goal of being a simple tool available for users. In Seninge et al.,^47^ a VAE was built to compress gene expression data to study gene modularity. The model was constructed with a linear decoder, with modules instead being supplied by the user to study cell regulation, as opposed to our work, where the gene modules are the desired output. An exciting study by Kuenzi et al.^48^ describes the DrugCell model, in which gene modularity was hard-coded into a neural network to predict drug outcomes in an interpretable latent space. While these studies used gene modularity in their modeling approaches, we note that prior knowledge can introduce bias.

Yet, it should be noted that the broader challenge of explainable AI has caught significant attention recently. There have been numerous efforts to design encoder-decoder architectures so that the representations in latent space can be interpreted.^49^ This has proven to be difficult, and, in our view, previous attempts in the machine learning community have been too general and have not considered the specifics of the molecular data. This remains a key challenge when using machine learning in a biological context.^50^^,^^51^ Our present work and that of others have shown a way forward in addressing this important challenge of understanding what machine learning models learn when trained on biological data. This challenge is now renewed in the recent context of the emergence of foundational models in bioscience based on single-cell genomics data.^52^

Our study is, in this sense, part of a growing approach to machine learning in life sciences that aims to capture the fundamentals of a system to make explainable predictions that accurately generalize outside of training data. Theodoris et al.^53^ used 30M single-cell transcriptomic profiles to understand the fundamentals of network biology and transferred these understanding features to context-specific predictions. Dalla-Torre et al.^54^ showed how a foundational model of human nucleotides could be used to predict molecular phenotypes. In a notable study by Lotfollahi et al.,^55^ single-cell data were mapped using a nonlinear encoder paired with a linear decoder and a biologically informed latent space to interpret gene processes. While Lotfollahi et al. presented intriguing results, we consider an entirely data-driven model that yields explanatory predictions to be a strength of our method.

The main scientific contribution of this study is the novel applicability of VAEs to extract useful information from the latent space representation and thereby enable an analysis of biological signals in human RNA-seq experiments. The model, together with the accompanying Python code, is freely available to users (see “data and code availability”), and we believe the software and the scientific insights will serve as a stepping stone toward moving analyses of high-throughput data into the AI era. Whereas our study focused exclusively on gene expression, extracting and studying cellular mechanisms in the latent space of a VAE are also applicable to other data, such as methylation, phosphorylation, and protein abundance data. We show how arithmetic operations on the latent space of a VAE can be used to extract and enhance disease signals, which we showcase by studying these disease signals from a disease module perspective. Furthermore, we also show how these vectors can be used to suggest candidate pharmaceutical compounds, which is an end goal of bioinformatic analyses. However, these approaches showcase the model only from a directly applied disease perspective, and we speculate that future applications can make use of the generative feature of VAEs in general, coupled with our suggested arithmetic operations related to disease signals, to study disease mechanics and offer possible treatments in novel, informative ways.

## Experimental procedures

### Data extraction and normalization

The GTEx was used as training data. We downloaded GTEx Analysis v.8 with the dbGaP accession phs000424.v8.p2, containing 17,381 healthy human RNA-seq profiles. We randomly partitioned the data into training (65%), validation (25%), and test sets (10%). To filter out less relevant genes without known functionality, we downloaded the STRING human PPI network^25^ and removed all genes that were not included in the STRING network, resulting in a total of 16,819 genes. This filtering approach aimed to reduce noise in the data by excluding genes with poorly understood interactions, leading to more robust and interpretable results We normalized the gene raw count values with only a logarithmic transformation, such that $X_{normalized}=In\left(x+1\right)$, a technique we and others have advocated.^9^^,^^45^ The Cancer Genome Atlas gene counts were downloaded in a preprocessed form from^19^ and processed in the same way as the GTEx data. For the GWAS data, we downloaded the “All studies” v.1.0.2 from the GWAS catalog and used the “REPORTED GENE(S)” column for the gene annotations. Moreover, we removed all traits with fewer than 100 associated genes. The KEGG gene pathway associations were downloaded from the Comparative Toxicogenomics Database.^56^ For the disease-control gene expression data we searched the Expression Atlas with the following inclusion criteria: the organism should be *Homo sapiens*, the technology should be RNA-seq mRNA, the type of experiment should be differential, it should be a case-control experiment, raw counts should be available, the sample tissue should be in our training data, and, for validation purposes, as implemented in Dwivedi et al.,^12^ there should be at least 100 known gene-disease associations present in the DisGeNET database. Moreover, we recently studied^24^ how upstream regulators of modules can be inferred using the data published by Elkjaer et al.^27^ and also included this case-control dataset in the analysis. The tissue-gene sets used in this study were sourced from the TISSUES 2.0 database, from which we selected curated known associations between gene expression and human tissue types.^20^ We matched the 30 distinct tissue types in our data with those present in TISSUES 2.0, considering only exact matches. Moreover, tissue-gene association in the database had to be supported by a minimum of 100 entries, and there had to be a minimum of 10 samples available for each tissue type in our test set. This process resulted in the identification of 16 distinct tissue types for further analysis. To further test against a wider array of tissues, we used the STRING. We downloaded the drug target data from https://go.drugbank.com/releases/latest#protein-identifiers using the “All” file.

### Model design and training

We implemented the model in the Python package Keras using a feedforward neural network structure. We designed our model based on the findings in our earlier study,^9^ which showed a robust model performance with respect to hyperparameter design. The encoder was built with one input node for each of the 16,819 genes in the data and a dropout rate of 20%. The hidden layers of the encoder had 128 and 64 × 2 hidden nodes, respectively, where 64 × 2 represents a mean and a log-transformed variation layer. The decoder was built to sample these mean and variation nodes and decompress the data to the full gene-set size. All nodes were implemented with the leaky ReLU activation function. The output layer was implemented with a linear activation function. The model was set to optimize the mean squared error between the linear activations and the normed gene expression data, assuming Gaussian noise in the data. The loss function is listed in Equations 1, 2, and 3. As such, L represents the loss of the model, being the weighted sum of the mean squared error (Equation 2), and the KL divergence loss (Equation 3):

(Equation 1) $$L=L_{\text{rec}}+L_{\text{KL}}$$

(Equation 2) $$L_{\text{rec}}=\beta \frac{1}{N}\sum_{i=1}^{N}∥y_{\text{true},i}-y_{\text{pred},i}{∥}^{2}$$

(Equation 3) $$L_{\text{KL}}=-\frac{1}{2}\sum_{j=1}^{D}\left(1+\log\left(\sigma_{j}^{2}\right)-\mu_{j}^{2}-\sigma_{j}^{2}\right)$$

In Equations 1, 2, and 3, $\mu_{j}$ and $\sigma_{j}^{2}$ are the mean and variance of the *j*-th dimension of the latent space, and *D* is the dimensionality of the latent space, in this case set to 64. Note that this model uses a squared loss for reconstruction rather than the negative log likelihood, which is typical in VAEs.

The initial training consisted of 500 epochs with the reconstruction error β multiplied by 100 while keeping the KL divergence error constant. Subsequent training consisted of 100 epochs, each with a diminishing reconstruction error multiplication factor β of 50, 20, and 10 until the KL divergence and reconstruction error were in equilibrium. The training used the National Academic Infrastructure for Super-computing in Sweden (NAISS), consuming approximately 20 core hours. The model is available for download at https://github.com/ddeweerd/VAE_Transcriptomics/.

### Latent space analysis

We analyzed whether and how different biological structures were represented in the latent space. To this end, we used RNA-seq data from the test set in the GTEx dataset and compressed it to the latent space. We hypothesized that the 64 μ-nodes of the latent space were not orthogonal and that vectors relating to biological processes would be linear combinations of the 64 μ-nodes. We used a linear PCA from the Python scikit-learn package to extract 64 vectors of node activation combinations and increased the node activations five times the standard deviation along each vector independently. Measuring the decompressed changes, we selected the top 500 changing genes of each vector and used a right-sided Fisher’s exact test for overlaps with genes in the KEGG, GWAS, and DrugBank data. The tests were performed over all 64 principal components. We assumed a background of 16,819 genes and used a Bonferroni correction of multiple testing, i.e., rejecting the null hypothesis where *p* <0.05/N, where *N* is the number of performed tests.

In the case of TF-target associations, we tested the direct relationship by increasing the activation values of input nodes pertaining to TFs and observed the change on the decoder level. We downloaded the TRRUST 2.0 TF-target associations and removed all genes not present in the 16,819 VAE input set and that had at least 10 associated target genes, yielding 190 TFs and 2,052 unique target genes. We again used the test data and calculated each gene’s variance and mean expression. We independently increased each TF’s expression with a factor of 5 standard deviations and passed this signal through the VAE. For each TF, we calculated the absolute change between the increased and the nonincreased mean test data across all genes. For each TF, we calculated the average increase in the known target genes divided by the average increase of genes not in the set of known target genes.

### Module extraction

Our module extraction method was implemented in two steps. First, we identified a vector corresponding to the difference between two states in the latent space. Next, we amplified and decompressed this vector to find its primary encoded gene expression variables.

The latent space vectors were derived from the latent space by compressing the case and control samples separately. For each node in the latent space, we calculated the average difference between the two factors. In detail, the mean activation vector **z** was calculated as the mean values of the μ-nodes inherent to a VAE. The vector **z** was calculated for each set, and the difference between disease and control activation vectors $\nu_{case-control}$ was calculated (Equation 4):

(Equation 4) $$\nu_{case-control}=z_{case}-z_{control}$$

Next, the vector $\nu_{case-control}$ was increased with a scalar factor η, with a default value of 3, and decompressed using the decoder *f*, as expressed in Equation 5. In Equation 5, $g_{augmented}$ denotes the decompressed gene expression vector from the compressed disease vector $\nu_{case-control}$. Notably, the inherent VAE property of continuous representation of features in the latent space is a prerequisite for this amplification to translate well into information on disease-relative gene changes after decompression:

(Equation 5) $$g_{augmented}=f\left(\eta *\nu_{case-control}\right)$$

To analyze $g_{augmented}$ with respect to the normal gene expression background, a matrix of 1,000 normally distributed random latent space variables, denoted as X, was decompressed to the background gene expression profile $G_{background}$, (Equation 6). We remark that $G_{background}$ is a matrix containing 1,000 random gene expression profiles:

(Equation 6) $$G_{background}=f\left(X\right);\;X∼N\left(0,1\right)\text{.}$$

Last, to estimate the most relevant genes in the vector $g_{augmented}$, we ranked each gene *i* for the number of times where the *i*:th gene, $g^{i}$, was larger than the corresponding *j*:th random sample for gene *i*. In other words, we counted the number of times the inequality in Equation 7 was satisfied and extracted the top ranking genes for further study:

(Equation 7) $$g_{augmented}^{i}>G_{background}^{i,j}$$

To filter genes when compared to the STRING PPI network with a confidence score cutoff of 700, we extracted the largest connected component subset of the genes and considered this set as the extracted disease module.

Since our study of tissue-specific genes had no case-control data, we generated 1,000 normally distributed random latent space variables and decompressed them to full transcriptomic profiles. The decompressed random samples were used as a control group, and the total number of samples in our GTEx test set for each distinct tissue type served as the case samples. Then, both the case and the control samples were compressed to the latent space, and the average activation of the control samples was subtracted from the average activation of the case samples, resulting in a latent space vector that, we hypothesized, encoded the difference between the two conditions. We decompressed this vector to a transcriptomic profile to investigate if relevant gene sets were captured in the latent space vector. Furthermore, we sampled an additional 1,000 normally distributed random latent space variables and decompressed them to full profiles for comparison.

In the analysis of disease modularity, we tested to what extent the output genes were more related than random chance. To this end, we used the STRING PPI network map at a threshold of 700, as implemented in the STRINGdb R package. The number of interactions between the genes in each disease dataset was compared to the general background of the STRINGdb, which we present as the median fold.

### Predicting pharmaceutical compounds

To quantitatively evaluate the association of the modules with drug targets listed in DrugBank, we conducted a right-sided Fisher’s exact test for each drug within the DrugBank database. In other words, for each disease module, we identified the DrugBank pharmaceutical compound with the highest overlap of associated drug targets. The results were ordered based on the false discovery rate (FDR)-corrected *p* values. The background set consisted of the overlap between protein products from our general background of 16,819 genes and the set of proteins found in DrugBank, resulting in a total background of 16,600 proteins.

## Resource availability

### Lead contact

For additional information or requests, please contact the lead contact, Rasmus Magnusson, at rasmus.magnusson@liu.se.

### Materials availability

All public data used herein are available at the respective original source.

### Data and code availability

The model, accompanied by relevant Python and R code, is available at https://github.com/ddeweerd/VAE_Transcriptomics/ at https://doi.org/10.5281/zenodo.13897260.^57^

## Acknowledgments

This work was supported by the Systems Biology Research Centre at the 10.13039/501100014791University of Skövde under grants from the Swedish Knowledge Foundation (grant 20200014 to R.M, Z.L.-P., and J.S.), 10.13039/100010769Petrus och Augusta Hedlunds Stiftelse (grant M-2023-2054 to R.M), the Assar Gabrielssons Fond (grant FB21-66 to R.M. and H.A.d.W.), and the 10.13039/501100004359Swedish Research Council (grant 2019-04193 to H.A.d.W. and M.G.). The computations were enabled by resources provided by the Swedish National Infrastructure for Computing (SNIC), partially funded by the 10.13039/501100004359Swedish Research Council through grant agreement no. 2022-06725.

## Author contributions

R.M., H.A.d.W., and Z.L.-P. conceived and designed the research. R.M. and Z.L.-P. supervised the analyses with contributions from all authors. H.A.d.W. conducted the computational analyses in collaboration with R.M. In addition, H.A.d.W., M.G., J.S., Z.L.-P., and R.M. were responsible for acquiring funding. R.M. wrote the initial draft with input from all authors.

## Declaration of interests

D.G. is an employee of Merck AB.

## Declaration of generative AI and AI-assisted technologies in the writing process

During the preparation of this work, the authors used ChatGPT-4 to improve the clarity and conciseness of parts of the text. After using this tool, the authors reviewed and edited the content as needed and take full responsibility for the content of the publication.

## Supplemental information

Table S1. The significant tissue matches

Table S2. The test statistics and number of associated genes in each case

Document S1. Figure S1 and Table S3

Table S4. The predicted drugs for each disease dataset
