# Supplementary material for: Latent space arithmetic on data embeddings from healthy multi-tissue human RNA-seq decodes disease modules
Source: Patterns (N Y). 2024 Oct 31;5(11):101093. doi: 10.1016/j.patter.2024.101093 (PMC11573900; doi:10.1016/j.patter.2024.101093)
Supplement: Document S1. Figure S1 and Table S3 [file mmc3.pdf]

Table S3. Odds ratios of identified disease genes in sets identified by different methods. Specifically, the odds ratio quantifies the degree of overlap between the genes identified in the VAE-derived module, those from Clique SuM, and the top differentially expressed genes (DEGs) with the corresponding disease-associated genes in DisGeNET.

Dataset	VAE	DEG	Clique SuM
Crohn Ileum 1	7.40	8.85	8.50
Crohn Ileum 2	10.60	7.21	6.95
Crohn Blood 1	2.78	2.99	4.14
Ulcerative Colitis Blood 1	2.42	3.71	4.15
Psoriasis Blood	3.65	2.58	8.13
Crohn Ileum 3	8.90	8.52	7.61
Crohn Ileum 4	11.70	7.74	8.83
Crohn Ileum 5	10.11	9.08	10.30
Crohn Ileum 6	4.19	6.89	7.25
Ulcerative Colitis Ileum 1	5.29	7.14	7.65
Crohn Ileum 7	8.28	6.13	3.48
Leishmaniasis Blood	4.83	1.74	5.44
Systemic Lupus Erythematosus Blood	0.66	3.49	3.84
Allergic Rhinitis Blood	1.47	2.38	5.65
Asthma Blood	1.63	3.00	5.26
Crohn Intestine	9.27	1.08	2.67
Ulcerative Colitis Intestine	7.38	6.55	6.58
Crohn Ileum 8	9.40	9.42	7.31
Sclerosing Cholangitis Colon	3.97	4.92	4.87
Ulcerative Colitis Colon	6.76	6.47	7.64
MS Active Lesion	10.22	5.64	4.92
MS Chronic Lesions	5.80	4.19	3.03
MS Inactive Lesions	9.40	3.56	3.06
MS Normal Appearing White Matter	7.15	4.82	4.56
MS Remyelination	6.28	3.19	4.46

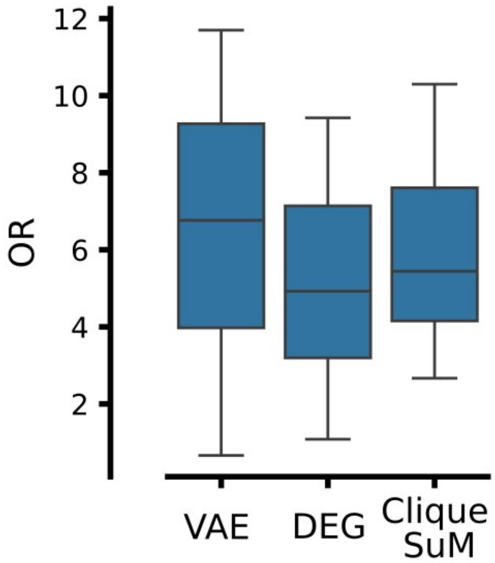

Figure S1: Odds ratios from Table S3, of identified disease genes in sets identified by different methods, shown in a boxplot.
